# Pitfalls and complications in the treatment of cervical spine fractures in patients with ankylosing spondylitis

**DOI:** 10.1186/1754-9493-2-15

**Published:** 2008-06-06

**Authors:** Christoph-E Heyde, Johannes K Fakler, Erik Hasenboehler, Philip F Stahel, Thilo John, Yohan Robinson, Sven K Tschoeke, Ralph Kayser

**Affiliations:** 1 Department of Trauma, Orthopedics, and Reconstructive Surgery, Charité, University Medical Center, Campus Benjamin Franklin, Hindenburgdamm 30, 12200 Berlin, Germany; 2 Department of Orthopedic Surgery, Denver Health Medical Center, University of Colorado, School of Medicine, 777 Bannock St, Denver, CO 80204, USA; 3 Department of Orthopaedics, Academic Hospital, Uppsala University, 751 52 Uppsala, Sweden

## Abstract

Patients with ankylosing spondylitis are at significant risk for sustaining cervical spine injuries following trauma predisposed by kyphosis, stiffness and osteoporotic bone quality of the spine. The risk of sustaining neurological deficits in this patient population is higher than average. The present review article provides an outline on the specific injury patterns in the cervical spine, diagnostic algorithms and specific treatment modalities dictated by the underlying disease in patients with ankylosing spondylitis. An emphasis is placed on the risks and complication patterns in the treatment of these rare, but challenging injuries.

## Introduction

Ankylosing spondylitis is a chronic systemic and inflammatory rheumatic disease with a variable course of the axial skeleton [1]. The disease manifests predominantly during the third decade, although early manifestation as of the age of 15 has been previously described [1-3]. Additionally there appears to be a latency of several years between the first manifestation and final diagnosis of disease [1,2,4]. Sacro-iliitis is usually the first clinical sign of ankylosing spondylitis. If a grade 2 bilateral or grade 3–4 ipsilateral sacro-iliitis is present, the diagnosis can be made with additionally one of the modified clinical New-York criteria (Table 1) [5]. Although a genetic predisposition is known, the exact cause of the disease remains unclear [1,6,7]. The strong association with HLA-B27, however, indicates the interaction with exogeneous factors (bacterial or viral infection) as a possible contributing factor [1,4]. An autoimmune response leads to the fibrosis and ossification of ligaments and joints of the spine, and in the end-stage to an individual and disseminated auto fusion of the spinal segments [8]. The corresponding classification by Hehne and Zielke is described in Table 2. This classification is widely used in Germany and relies on radiographic findings of syndesmophytes and the grade of ossification of the anulus fibrosus and the facet joints. This classification is helpful for the decision making regarding the modality of surgery in case of a necessary correction [9].

**Table 1:** The modified "New-York-Criteria" [1,5].

**Clinical Criteria:**
Lower back pain and stiffness for more than 3 months with improvement after exercise, but not with rest
Reduced motility of the lumbar spine in the sagittal and frontal axis
Restriction of the chest expansion (age and gender related)
**Radiological Criteria:**
Sacroiliitis at least grade 2 bilateral or grade 3–4 ipsilateral

**Table 2:** Hehne und Zielke classification in ankylosing spondylitis [9].

**Type**	**Definition**	**Syndesmophyte characteristics**	**Synonymes**
**I**	Dorsal ossification	Simple ossification of the dorsal spine bodies, no syndesmophytes	Spondylathritis type
**Iia**	Incomplete anular ossification	Tender, the anulus fibrosus ventrally with or without lateral syndesmophytes, without overlapping of the vertebral bodies (incomplete)	Anulus-type
**Iib**	Complete anular ossification	As type IIa, but with syndesmophytes overlapping the vertebral disc (complete)	Anulus-type
**IIIa**	Partial ostotic ossification	Thick and wide syndesmophytes, often with cortical- and spongiosa structure, corresponding the bamboo-type, but not present in all segments (incomplete)	Ligament/sub-ligament type
**IIIb**	Total ostotic ossification	As type IIIa, but several thoracic and lumbar segments are affected	Bamboo-spine

Resulting kyphosis, spinal rigidity and secondary osteoporosis lead to changes of the biomechanical characteristics of the spinal column [10-13]. Secondary to kyphosis, the ventral displacement creates pathological tension and shearing forces to a spine with lacking flexibility [3,12]. Furthermore, the reduced muscle activity and increased muscle degeneration causes an overall loss of muscle strength [11]. The resulting posture change, with its pathologically elongated lever arms and reduced bone quality, can lead to serious injuries even after minor trauma mechanisms [3,6,8]. Frequently, no trauma is recalled by the patient, but rather an abrupt motion or other inadequate mechanisms [3,6,14]. Especially if a complete rigidity of the spine is present, i.e. Hehne and Zielke type IIb, III and IIIb (Table 2), there is an increasing risk sustaining such injuries [9,12]. In these cases, the rate of neurological complications is extremely high, and special attention must be addressed to delayed or secondary neurological deterioration [3,11,14-16]. Neurological complications, however, are mostly seen in fractures with complete disruption.

Operative management of the injured cervical spine with ankylosing spondylitis is difficult, technically challenging and associated with a high complication rate [12,14,16,17].

### Epidemiology

The prevalence of ankylosing spondylitis ranges between 0.2% and 0.55% [18,19]. Similar to the low prevalence of ankylosing spondylitis, vertebral fractures in case of ankylosing spondylitis represent a rare entity [4]. Fractures of the cervical spine represent the most common level of injury in this patient population [4,7,20]. Centers with a high case load of spine trauma and a solid experience in the management of ankylosing spondylitis still have a low incidence of such injuries. Published series have usually been accumulated over a longer period of time [2,3,8,12,14,16,20,21]. A questionnaire in 1071 patients with ankylosing spondylitis revealed a 5.1% prevalence of vertebral fracture history. Up to 14% of patients with ankylosing spondylitis will experience a clinically manifest vertebral fracture during their lifetime [22]. A summary of published case series found cervical fractures to comprise 73% of vertebral fractures in ankylosing spondylitis (n = 130) [18]. Sixty-five percent of patients with vertebral fractures in ankylosing spondylitis had neurological deficits [18]. The drawing attention to these injuries is a result of the specific and uncommon fracture configuration with sometimes grotesque dislocations, the high rates of neurological complications and the challenging surgical management. The male to female ratio is 2.5:1 in the prevalence of the disease, and the incidence of cervical spine injuries is higher in males [3,8,12,14,16,20]. One study reported a prevalence of vertebral fractures of 6.2% among males and 4.6% among females [22]. Most studies report an average age of 60 years or slightly higher [14,21,23]. Affected patients usually have a long disease progress and a "peak" during the second or third decades after their initial diagnosis [24]. The average disease duration at the time of vertebral injuries was 24.0 ± 11.5 years after onset [22]. Injuries are mostly localized in the lower cervical spine (C5/6 and C6/7) and in the cervico-thoracic junction, although any area of the cervical spine may be affected [3,8,20,25]. Furthermore, cervical instability secondary to rheumatoid destruction, has been observed in the upper cervical spine. These changes have to be considered both in the diagnostic work-up and the therapeutic approach [8,26]. Simple falls, followed by motor vehicle accidents and high energy trauma, are among the most common mechanisms of injury of the cervical spine in these patients [3,20].

### Classification

The spinal rigidity with its atypical and complex vertebral fractures makes the classical column model for fracture classification [27,28] hardly applicable in ankylosing spondylitis patients [12,16]. Metz-Stavenhagen et al. [12] classified lesions of the cervical spine into two major groups (Table 3). The first is comprised of complex fractures of all vertebral structures at the lesion level. This type of injury combined with the high leverage from the stiffened spine may result in severe posture errors and dislocations. By classification according to Magerl et al. [27], such fractures are described as flexion-/distraction or hyperextension injuries, with the latter occurring most frequently [13,14,20,21,25]. This classification, which was originally designed for thoracic and lumbar fractures, can be adapted to the lower cervical spine [29]. According to Cornefjord et al. [21] fractures of the cervical spine in ankylosing spondylitis are usually diagnosed as hyperextension injuries caused by the spinal deformation when the patient is in the supine position. Furthermore, the authors postulated that a portion of these cases could be posture errors acquired secondarily occurring after a primary flexion injury. The fracture line may pass either through the disc space or through the vertebral body, although fractures of the ossified discs are more frequent [20]. The often incomplete ossification of the nucleus pulposus has been proposed as a possible cause for the latter described fracture pattern [13,30]. The rate of neurological complications including complete paraplegia is disproportionately high [12,15,20,25]. Fractures, particularly when caused by minor trauma, can be localized in the anterior part of the spine exclusively, with the imminent danger of secondary fractures of the dorsal structures under stress [17]. On the other hand stepwise sintering of the vertebral bodies has been described, leading to consecutive dislocation or resulting in significant deformities [12].

**Table 3:** Fracture classification in ankylosing spondylitis according to Metz-Stavenhagen et al. [12].

**Classification**	**Description**
**Type 1**	Complex fracture pattern involving anterior and posterior – bony and ligamentous structures of the spine at the level of injury
**Type 2**	Consecutive sintering

### Diagnosis

**Figure 1:**
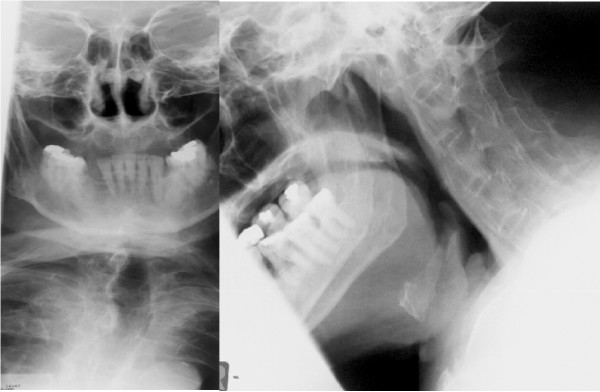
X-Ray's in standard plain show a reduced view of the lower cervical spine and of the cervico-thoracic junction. Furthermore, an accurate evaluation is difficult due to the ossification and osteoporosis.

**Figure 2:**
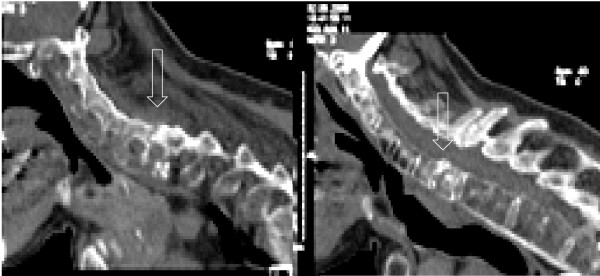
The CT 2-D reconstruction shows a thin fracture line in a completely but not dislocated fracture at C6/7.

The radiological imaging of spine fractures in patients with ankylosing spondylitis demands special attention since this injuries are frequently missed and delayed in their diagnosis [10,12-14,20,21]. Basic imaging consists of plain X-rays of the cervical spine in two planes. Often additional views of the occipito-cervical and cervico-thoracic junctions are required. Adequate assessment of these plain films can be very difficult. The rigid elevation of the shoulders prevents adequate imaging at the cervico-thoracic junction (Figure 1) [7,10,31]. Furthermore, osteoporotic changes make visualization of fractures more difficult, particularly in the presence of thin fracture lines. The latter, without any primary dislocation, can be missed in plain films. A thin-cut (2 mm) multislice CT with 2-D reconstructions is the most sensitive strategy to visualize these fractures (Figure 2) [3,32,33]. Broader slice imaging may not allow adequate fracture visualization [32]. Further information may be gained from 3-D CT reconstructions [7]. An MRI may be indicated for the evaluation of ligaments, intervertebral discs and the myelon. A fresh edema of the vertebral body, in terms of a bone bruise, may represent an indirect sign of a hidden injury [7,17]. The MRI furthermore represents the most sensitive tool to visualize an intraspinal bleeding, which can occur in these patients due to the solid fixation of epi- and peridural veins with the fibrotic or ossified tissue [11,20].

Asymptomatic or mildly symptomatic multi-level spinal injuries are more often described in the literature. Therefore, it is recommended to search carefully for associated cervical spine injuries and to radiologically clear the entire spine [8,13,14,20,34]. Scintigraphic bone scans can be helpful for screening a patient, especially if he cannot be placed in the MRI due to the spinal deformity. Nevertheless scintigraphy looses its diagnostic value ten days after the initial trauma [12]. Furthermore, scintigraphy is not specific for fractures and is therefore not the first choice in the acute phase [20,32,35].

On plain radiographs it can be difficult to distinguish between fractures and the typical inflammatory discitis known as "Anderson-lesion". In these cases, MRI allows their differentiation [12,36].

### Therapeutic options

The primary goal of treatment is bony healing without significant loss of reduction. The literature provides recommendations for both non-operative and operative treatment. Indications for operative treatment are based on the criteria for instability, incorrect posture, pain and neurological deficit [12]. Frequently, a Halo external fixator is recommended for the initial stabilization if a planned surgical treatment has to be delayed [20]. The topic of whether surgical correction of pathological spinal posture is truly relevant remains controversial. This is of special interest in fractures of the cervical spine and the junctional areas [12,20]. Spinal correction thought to be secondary to the main therapeutic goal of fracture stabilization [2,6,20].

### Non-operative treatment

Non-operative treatment strategies can only be successful if an adequate stabilization and realignment of the cervical spine are achieved in all planes. For this purpose, an external fixation with the Halo is usually required. The expected duration of treatment until fracture healing is somewhere between ten to sixteen weeks [20]. Because of the often highly unstable fractures, the osteoporotic bone, compromised skin quality, and pathological chest rigidity, Halo treatment is associated with a high complication rate and a high risk of non-unions [6,8,11-14,23]. Loss of reduction, non-union and neurological deterioration have been reported after non-operative treatment, which often leads to secondary surgery [3,20,30]. In older fractures with consecutive severe kyphotic deformities, primary non-operative treatment can be advisable. In these cases, the Halo fixator allows gentle and stepwise correction prior to definitive operative fixation [12,20]. History recommendations suggested treatment in traction [2,30,37]. According to recent publications and also to our own experience, such treatment strategies are not to be recommended due to the associated long-term immobilization and to complications related to bed rest and to Halo fixation [3,12,14,20].

### Surgical treatment

The primary aim of surgical treatment is the maintenance of fracture realignment with adequate stabilization measures until the bone has healed completely. Decompression of spinal stenosis may be performed in the same operative session. This may be achieved through a single anterior, single posterior or a combined one-time or staged posterior-anterior or anterior-posterior approach. Additional iliac crest bone grafting or the implantation of titanium cages with cancellous bone graft may be necessary for the anterior approach. By our own experience, the choice of the specific surgical approach does not only depend on the individual fracture pattern, the bone quality, co-morbidities and associated fractures, but also on the surgeon's personal preference. The anterior or posterior-only approaches have the benefit of short operative times and reduced postoperative morbidity [12], but combined approaches provide higher primary stability and offer the option for possible aftercare in a soft C-spine collar [11,13,20]. The anterior-only stabilization is biomechanical inferior to the posterior-only stabilization, and especially to the combined stabilization techniques [38,39]. Nevertheless, there is no evidence above expert opinion supporting one or the other stabilization method (Table 4). Spinal decompression is needed in addition to fracture stabilization to address spinal stenosis, free bone fragments in the spinal canal, and epidural bleeding or spinal cord edema [8]. Most authors agree on the need for long instrumentations to guarantee sufficient load sharing in osteoporotic bone. This strategy, however, is irrelevant to the function due to the biomechanical loss caused by the primary disease [8,11,12,20,23]. In this context, angular stable implants and screws as well as the combined use of cement and screws are recommended due to their increased pull-out strange [14,40]. The literature points out a higher risk for epidural bleeding, which usually is considered in spine surgery in this patient group [6,16,41]. A possible cause may be the difficult positioning of the patient with increased intraabdominal pressure and consequent venous dilation while on the surgical table [6,19,42]. The pathological fixation of epi- and peridural veins within the fibrotic or ossified tissue may be another reason for this phenomenon [11,20]. Measures to minimize bleeding, such as autotransfusion and permissive hypotension are standard requirements [6,41].

**Table 4:** Key studies related to surgical stabilization of cervical spine fractures in ankylosing spondylitis.

**Authors**	**Year**	**Design**	**N**	**Preoperative neurological compromise**	**Surgical stabilisation**	**Outcome of stabilization**
Olerud et al. [16]	1996	Retrospective case series	17	?	Anterior and posterior stabilisation	?
Taggard & Traynelis [23]	2000	Prospective case series	7	3	Posterior	All survivors with solid fusion after 3 months
Guo et al. [45]	2004	Retrospective case series	11	8	Anterior and posterior	All survivors with fusion at final follow-up
Cornefjord et al. [21]	2005	Retrospective case series	19	8	Posterior and combined	Fusion in all patients
Zdichawski et al. [3]	2005	Retrospective multicentric case series	19	?	Anterior, posterior, and combined	3 cases with implant failure (all cases anterior-only)
Payer [46]	2006	Retrospective case series	4	2	Anterior, posterior and combined	Fusion in all survivors
Einsiedel et al. [14]	2006	Retrospective multicentric case series	37	36	Anterior, posterior and combined	5 cases with early implant failure (all cases anterior only)

#### • Anesthesia/Positioning

**Figure 3:**
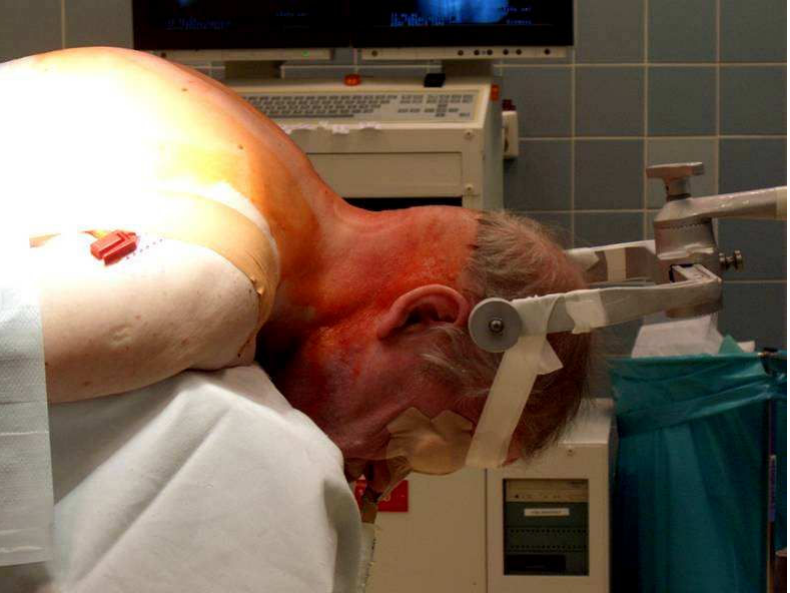
Positioning of a patient with ankylosing spondylitis on the surgical table is technical demanding.

Patients with ankylosing spondylitis are at increased risk for posture deterioration and iatrogenic fractures of the spine during the surgical procedure, especially while under sedation and anesthesia [17,20]. A detrimental consequence is the worsening of neurological impairment [6,17,20]. This holds true for the preclinical phase throughout the rehabilitation. The acute onset or progression of neurological deficits had been reported after emergency intubations [8]. To avoid further motion to the cervical spine, intubation should be performed fiberoptically [20]. Lu et al. [43] reported successfully performed „blind intubations“ using a laryngeal mask for guiding the tube in patients with ankylosing spondylitis in elective procedures. During any transportation or positioning, special attention must be addressed not only to the pathological posture but also to the altered spine biomechanics. During the positioning procedure, the OR-table must be adapted to the patient's posture and the patient correctly placed to avoid any possible pressure sores (Figure 3). This positioning is highly demanding and must take place under secured protection of the cervical spine. If no Halo fixator was used previously, final positioning of the cervical spine must be performed with the use of lateral C-spine flouroscopy [6]. Patients with ankylosing spondylitis are at high risk during all transfers, positioning procedures and handling during anaesthesia and surgery [6,11,20].

#### • Anterior stabilization

Anterior stabilization may be ideally performed in patients with good bone quality, where the fracture can be precisely reduced and perfect axial alignment can be obtained [3,12]. With an anterior approach, the feasibility for long-distant fixation is limited. As a primary measure before the patient is put into prone position it secures the spine and reduces the risk for a neurological complication during positioning. In cases of severe deformity, the anterior approach to the cervical spine may not be possible. The anterior-only stabilization is rarely performed due to the aforementioned conditions. Further, posterior and combined approaches demonstrate a higher biomechanical stability [3,12,38,39].

#### • Posterior stabilization

Posterior stabilization is recommended for unstable fractures with the risk of possible translation and with a sufficiently stable anterior column [6,23]. Fox et al. [8] recommend the use of a Halo fixator after posterior stabilization, until fracture consolidation is radiologically documented. Other authors demonstrated good results without a Halo fixator after an extended posterior stabilization [20,23]. Improved stability of the posterior rod and screw fixation was described after the use of cervical pedicle screws compared to lateral mass screws [20,38].

#### • Combined 360°-fusion

**Figure 4:**
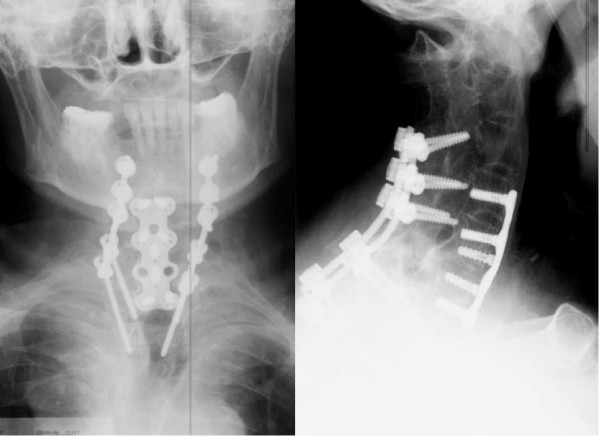
Same case of a 64 years old patient showed in figure 1 and 2. A one-time ventro-dorsal surgery was performed, with ventral plating and a dorsal instrumentation.

**Figure 5:**
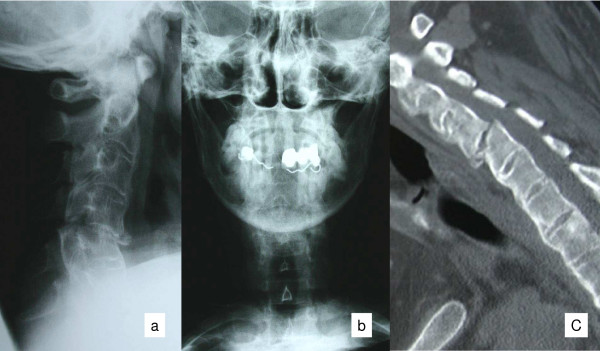
A 44 years old patient with ankylosing spondylitis and a complete fracture of C 3/4 and partial dislocation (panels a, b and c).

**Figure 6:**
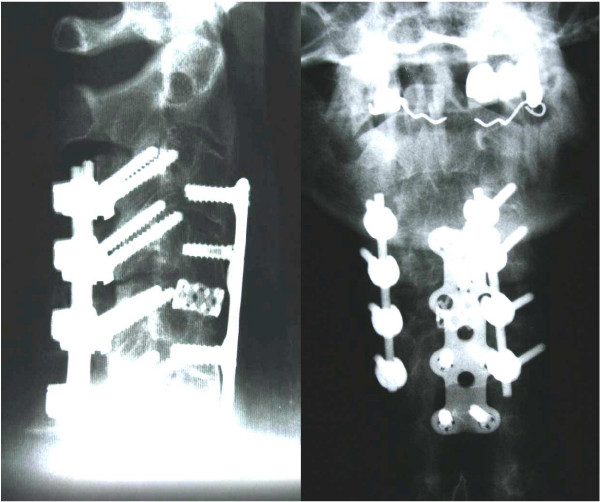
Same case of the patient showed in figure 5. A well reduced cervical spine is visible after a one-stage ventro-dorsal spondylodesis and ventral cage implantation for a defect.

Combined posterior-anterior or anterior-posterior stabilization is recommended for unstable injuries with translation or defects of the anterior column with an additional kyphotic deformity (Figure 4) [3,11,12,14,16,23]. Surgery should begin primarily from the side where the fracture can be best reduced, usually from anterior [20]. Fracture type and the patient's general condition will determine a one-time or staged surgery [14]. Additional anterior bone-grafting with a solid iliac crest graft or with a cage filled with cancelleous bone, might be necessary for bigger defects (Figure 5 and 6) [20,34]. Due to the greater stability, several authors recommend this combined approach as the standard procedure [3,14,16,20].

### Postoperative Rehabilitation

During aftercare, patients have an increased risk of developing severe complications due to their preexisting rheumatoid disease [3,44]. The reduced pulmonary function caused by kyphosis, chest rigidity, reduced diaphragmatic expansion and the potential presence of fibrosis increase the risk for pulmonary infections. This represents the main cause of post-operative mortality [6,8]. Hence, fractures need to be stabilized in a way that allows an aggressive early post-operative mobilization.

The reduced skin resistance, attributed to the rheumatic disease, can provoke the formation of pressure sores even in the area of the rigid cervical collar [14]. Attention should be paid to the Halo fixators for possible pin tract infection. The pins of a Halo fixator are also at an increased risk of loosening in case of osteoporotic bone quality [3].

All of these problems may lead to increased morbidity and mortality in the postoperative treatment [2,6,13,20]. Olerud et al. [44] described an increased complication rate in elderly patients and in patients with preexisting neurological deficit. At the same time, the mortality rate for ankylosing spondylitis patients with a cervical fracture is higher than in a comparison group with only a cervical fracture. The main goal, therefore, is an early postoperative mobilization without the aid of Halo fixators. In our opinion, a long instrumentation with a combined dorso-ventral 360° fusion is the best option that allows early postoperative mobilization of the patient with ankylosing spondylitis.

## Discussion

As outlined in this article special attention required in patients with ankylosing spondylitis with suspected cervical fractures. Due to the low prevalence of this particular underlying disease in the general population these injuries are a rare finding [4,7]. Injuries of the cervical spine of these patients may occur even after minor traumas, and in certain cases, patients do not recall any trauma [3,6,8,12]. The rigidity of the spine, the resulting kyphotic deformity, the associated osteoporosis, and the degenerated stabilising muscles lead to an increased vulnerability of the spinal column. The resulting prolonged lever arm influences the evident pathological posture [12,14,16,20]. Therefore, a meticulous screening of the entire spine is necessary in these patients with new onset or increased pain, posture alterations of the head or change in forward gaze [8,13,14]. The required checkup should not only include the clinical and neurological examination, but also a standardized radiological work-up. The x-ray validity, however, is often reduced because of the morphological specifics, osteoporosis, and the often unrecognizable thin fracture line in non-displaced fractures [7,10,31]. Therefore, a thin-cut (2 mm) multislice CT with 2-D reconstructions of suspicious areas is strongly recommended. Further, an MRI is necessary for unclear statements and for questionable situations, such as spinal canal compromise with neurological deficits [6,11,17,20,32,33].

Injuries of the lower cervical spine and the cervicothoracic transition prevail in the cervical spine [12,20,25]. Complex fracture patterns with severe dislocation and neurological impairment are to be found above average [2,14,16,20].

As outlined above, two distinct classification systems have been proposed for cervical fractures in patients with ankylosing spondylitis. The standard classification by Magerl and colleagues [24] is of limited use for this particular patient group, due to significantly altered anatomic morphology. The classification by Metz-Slavenhagen [12] considers complete disruption of the entire spine and consecutive sintering. Thus, incomplete fractures are missed by this classification scheme. Due to the weaknesses of both classification systems for patients with ankylosing spondylitis, we recommend the additional descriptive characterization of individual fracture patterns.

The main goal of treatment is the stabilization of the fracture in the correct position until complete fracture healing is achieved. Surgical procedures offer advantages compared to non-operative treatment options as mentioned above [12,14,20]. There is no consensus in the literature regarding on favorable operating technique. Isolated anterior stabilizations are rarely indicated [12]. The frequently recommended posterior-only instrumentation results in higher stability opposed to the single ventral stabilization, especially using angle-stable devices [6,21,23]. The combined anterior-posterior or posterior-anterior approaches are preferred due to the resulting higher primary stability. To start surgery in prone position reduces the danger of neurological deterioration during positioning and allows a first reduction and stabilization in a safe way [3,11,12,14,16,20]. Agreement is obtained on the need for long instrumentation in order to obtain a better distribution of the resulting forces at the bone-implant interface [3,11,12,14,20,23].

Due to its increased vulnerability, the spinal column of patients with ankylosing spondylitis is compromised during every therapeutic procedure [17,20]. This includes the transport and positioning of the patient, as well as anesthesia, surgical procedures and the postoperative rehabilitation. Therefore, a standardized management is required in patients with ankylosing spondylits, including fiberoptical intubation, appropriate transport maneuvers and positioning under the avoidance of any brusque manipulation. Other relevant items are consequent vital sign monitoring, periodic neurological checkups and thorough examinations into newly occurring symptoms [6,8,20,41,42]. A stable and safe surgical stabilization which allows immediate postoperative mobilization remains a main goal of treatment. Therefore an appropriate standardized workup, the consideration of specific aspects regarding both the underlying disease and the particular risk of the biomechanically altered spine are of high importance for an adequate outcome in this special patient group. [2,3,6,12,14,20,23,44].

## Competing interests

The authors declare that they have no competing interests.

## Authors' contributions

CEH and RK developed and realized the idea of this review with particular concern to pitfalls and complications, CEH wrote and RK and YR revised the manuscript. JKF, TJ, SKT and YR analyzed the patient series in Berlin regarding complications and specifics. Furthermore, JKF, TJ, SKT and YR sampled and analyzed the literature, EH and PFS did it in the same way in Denver. In addition, EH and PFS corrected the manuscript and contributed input regarding the manuscript form. All authors read and approved the final manuscript.
